# Diagnosis and Treatment of Pseudo-Class III Malocclusion

**DOI:** 10.1155/2014/652936

**Published:** 2014-11-24

**Authors:** Ariel Reyes, Luis Serret, Marcos Peguero, Orlando Tanaka

**Affiliations:** ^1^School of Health and Biosciences, Pontifícia Universidade Católica do Paraná, Brazil; ^2^Universidad Intercontinental, México, Mexico; ^3^Private Practice in Santo Domingo, Dominican Republic; ^4^Pontifícia Universidade Católica do Rio de Janeiro, Brazil; ^5^Graduate Program in Orthodontics, School of Health and Biosciences, Pontifícia Universidade Católica do Paraná, Rua Imaculada Conceição 1155, Bairro Prado Velho, 80215-901 Curitiba, PR, Brazil

## Abstract

Pseudo-Class III malocclusion is characterized by the presence of an anterior crossbite due to a forward functional displacement of the mandible; in most cases, the maxillary incisors present some degree of retroclination, and the mandibular incisors are proclined. Various types of appliances have been described in the literature for the early treatment of pseudo-Class III malocclusion. The objectives of this paper are to demonstrate the importance of making the differential diagnosis between a skeletal and a pseudo-Class III malocclusion and to describe the correction of an anterior crossbite. The association of maxillary expansion and a 2 × 4 appliance can successfully be used to correct anterior crossbites.

## 1. Introduction

Class III malocclusion was originally described by Angle as a condition in which the relationship of the jaws is abnormal and all of the mandibular teeth occlude mesial to normal by the width of one bicuspid or more [1]. The etiology is associated with environmental and genetic factors, and a higher incidence has been observed in an Asian population [2]. The etiological factors of this malocclusion have been classified into three groups: (a) functional, which includes abnormal tongue position, nasal-respiratory problems, and neuromuscular conditions; (b) skeletal, such as during maxillary transversal deficiency; and (c) dental, which includes ectopic eruption of the maxillary central incisors and early loss of the deciduous molars [2, 3].

Pseudo-Class III malocclusion is characterized by the presence of an anterior crossbite due to a forward functional displacement of the mandible. In the mixed dentition, the mesial step cannot exceed 3 mm, the maxillary incisors present retroclination, and the mandibular incisors are proclined and spaced [3, 4]. When patients are guided into a centric relationship, they usually show an end-to-end incisor relationship involving the performance of a forward functional mandibular shift due to a muscular reflex so that the posterior teeth are able to occlude. It is for this reason that this type of malocclusion has been described as a pseudo- or functional Class III malocclusion [2, 3, 5, 6].

In most cases, retroclined maxillary incisors are the main cause of pseudo-Class III malocclusion [6]. Often, a molar Class I relationship is present with a normal mandibular appearance and a straight facial profile, disguising the skeletal discrepancy that may exist [2]. However, patients with skeletal Class III malocclusions show a posterior crossbite and maintain their molar relationship when guided to a centric relationship [3]. Correction of the anterior crossbite must be carried out as soon as it is detected to increase the orthopedic effects, thereby increasing the long-term stability of the treatment results [3].

## 2. Case Presentation

**Figure 1:**
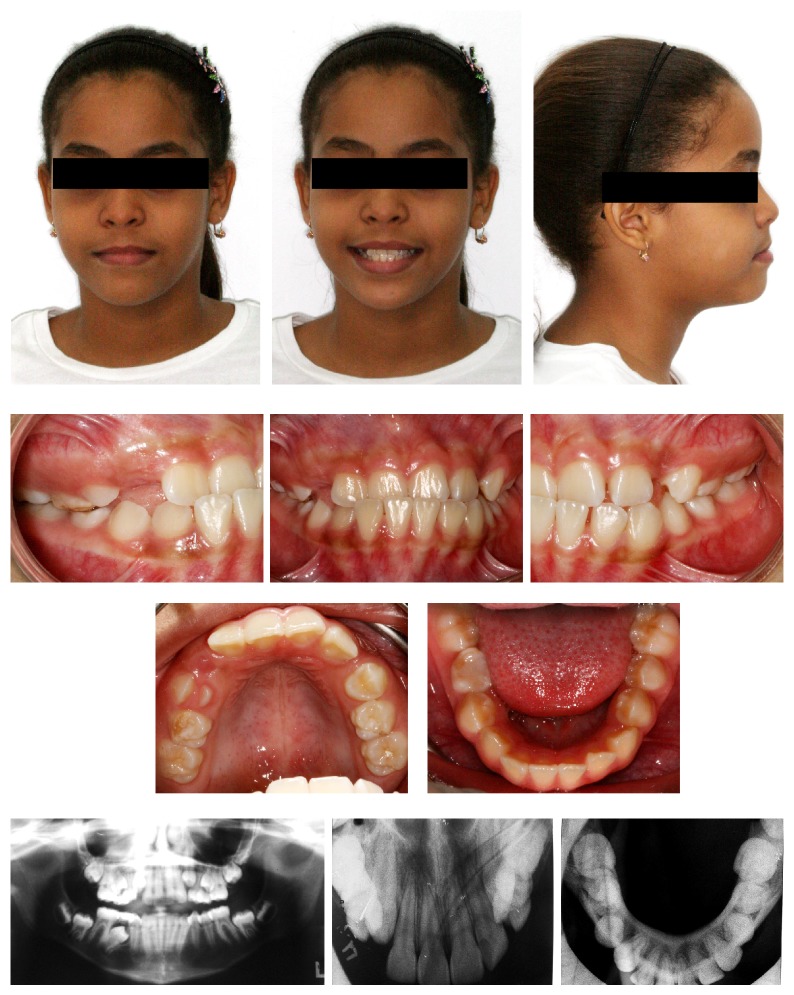
Pretreatment photographs.

A 10-and-a-half-year-old girl was referred by her dentist with the following chief complaint: “My mandible is forward and my upper teeth look ugly.” The extraoral facial examination revealed a straight profile, lower lip protrusion, and a dolichofacial pattern. The intraoral evaluation revealed late mixed dentition due to the presence of both the deciduous maxillary second molars and the deciduous mandibular right second molar; the absence of maxillary deciduous canines due to prior extraction; a Class I molar relationship; anterior crossbite of the maxillary central and lateral incisors; crowding in both arches; and a lack of space for the maxillary canines to erupt (Figure 1).

**Figure 2:**
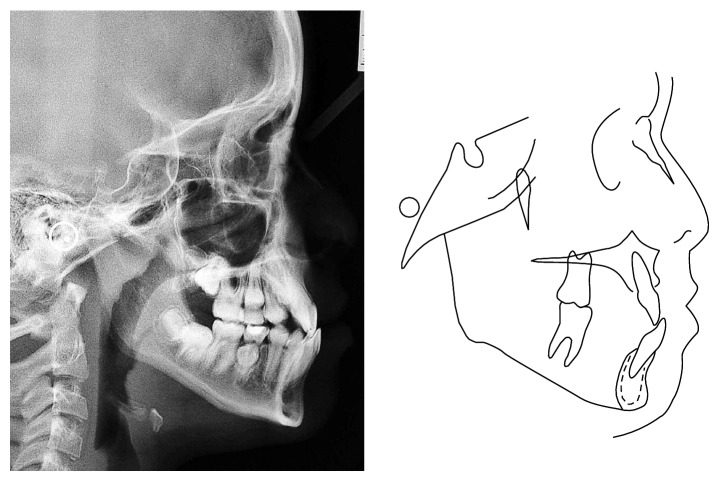
Lateral cephalogram radiograph.

The panoramic radiograph revealed that the mandibular right second premolar was mesially angulated and that the eruption sequence was favorable, and an occlusal radiograph indicated that the tooth was actually in a transalveolar position with the crown located lingually. The cephalometric analysis revealed a Class I skeletal relationship (ANB = 2°), a clockwise growth pattern (SN.GoGn = 40°, FMA = 29°), protrusion of the mandibular incisors (IMPA = 99°, 1.NB = 35°), retrusion of the maxillary incisors (1.NA = 18°, 1-NA = 1 mm), and protrusion of the lower lip (Ricketts E-line = 3 mm) (Figure 2, Table 1). Based on these diagnostic findings, it was concluded that the patient presented a skeletal Class I relationship.

The objectives were to maintain the Class I molar relationship, correct the anterior crossbite, and augment the maxillary arch perimeter, allowing guided eruption of the maxillary canines and orthodontic traction of the mandibular right second premolar while taking advantage of the E-space.

The diagnosis of skeletal Class I improved her prognosis, and correction of the anterior crossbite was attempted through maxillary expansion associated with a fixed 2 × 4 appliance. Other options included the following: (1) a removable appliance with a Z-spring to procline the maxillary incisors labially, (2) an angulated bite plane, and (3) functional appliances, although the lack of cooperation of some patients and the inability of the appliances to promote correct alignment and leveling are the biggest disadvantages of these appliances. To achieve good alignment and leveling, a fixed appliance must be used [2, 5]. Using a facial mask was not considered because of the age of the patient and the fact that the harmonic basal bone relationship was within normal limits.

## 3. Treatment Progress

**Figure 3:**
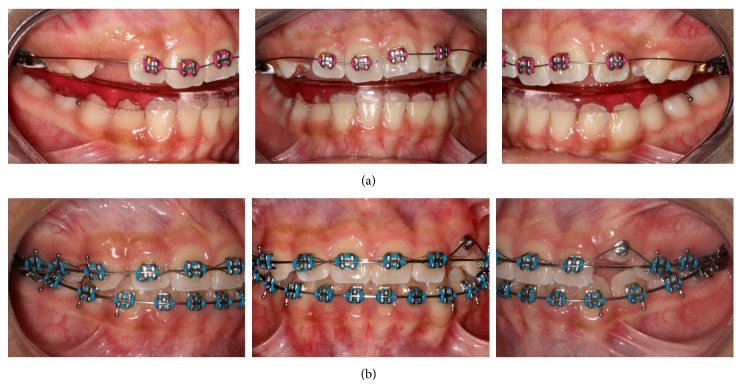
(a) Maxillary 2 × 4 associated with a mandibular bite plane; (b) treatment progress 0.017′′  × 0.025′′ SS.

The patient was first submitted to a rapid maxillary expansion, once finished this first phase we bonded a preadjusted Edgewise 0.018′′ slot 2 × 4 appliance with an initial 0.014′′ NiTi arch wire in the maxillary arch associated with a removable bite plane in the mandibular arch. After correcting the anterior crossbite, the use of the bite plane was suspended, and maxillary sequential bonding was performed visualizing a corrective orthodontic treatment in the second phase. A heat-activated 0.016′′ × 0.022′′ NiTi arch wire was placed as initial arch, followed by a superelastic 0.017′′  × 0.025′′ NiTi arch wire. The treatment of the mandibular arch began two months after inserting the maxillary 0.017′′  × 0.025′′ NiTi arch; the arch wire sequence in the mandibular arch followed the same pattern. Both arches finished with a 0.017′′  × 0.025′′ SS (Figures 3(a) and 3(b)).

**Figure 4:**
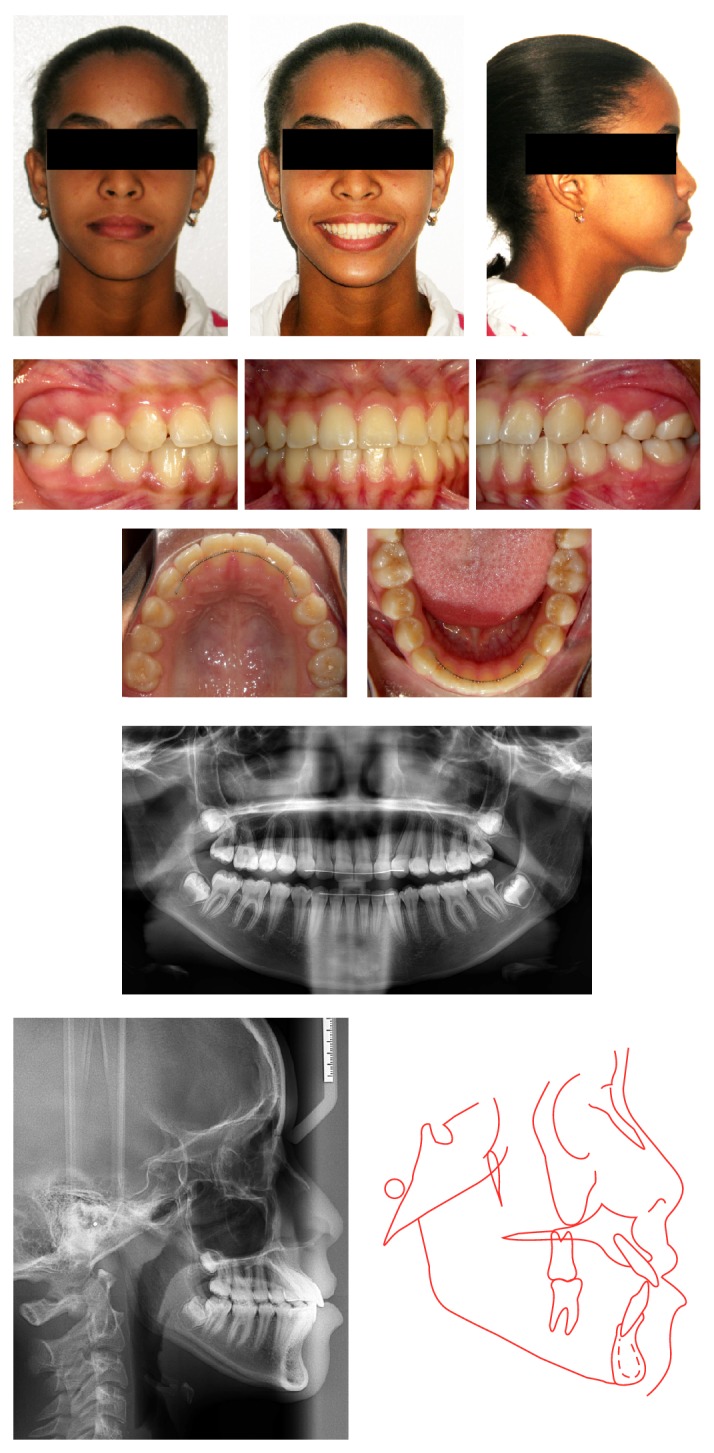
Posttreatment photographs and radiographs.

At the end of the treatment the pseudo-Class III relationship was compensated during the second phase. The space gained with the maxillary expansion and maxillary incisors protrusion helped in the eruption of the maxillary canines and the correction of the anterior crossbite. In the mandibular arch, the position of the transalveolar right second premolar self-corrected and erupted after extraction of the mandibular deciduous right second molar, avoiding the need for the surgical exposure planned at the beginning of treatment. Facially the treatment did not change her growth pattern, and the Class III characteristics were maintained (Figure 4).

## 4. Discussion

Treatment of a pseudo-Class III malocclusion must be performed as soon as it is detected and should be considered as a Class III malocclusion [4]; however, the clinician is unfortunately not always able to evaluate the patient during the developmental stage of this type of malocclusion. Anterior crossbite has been associated with a variety of complications, such as gingival recession of the lower incisors, incisal wear, and worsening of the growth pattern; correcting an anterior crossbite consequently increases the maxillary arch perimeter, offering more space for the canines and premolars to erupt and therefore a more stable orthopedic result [4–8].

The functional appliances used to treat Class III malocclusion work by permitting the eruption of the maxillary molars and maintaining the mandibular ones in position, leading to an occlusal plane rotation that helps shift the molar relationship from Class III to Class I [9]. Face mask protraction creates a counterclockwise rotation of the maxilla and a clockwise rotation of the mandible while increasing the inferior facial height and turning the patient's profile more convex [4].

When treating young patients with anterior crossbite in mixed dentition, better results can be achieved through the association of maxillary expansion due to orthopedic stability and the movement of the maxilla down and forward [10]. In 84% of cases, a self-correction could be expected without the need for any other type of appliance [11]. The association of maxillary expansion with fixed appliances improves the arch perimeter, reducing the number of extractions in patients with slight to mild crowding. The increase has been quantified to span up to 6.0 mm in the maxillary arch [12]. Other advantages of fixed appliances include better three-dimensional control of the tooth and the release of continuous forces [8]. Our patient benefitted from this combination because the space gained with the maxillary expansion and incisors protrusion helped for the eruption of the canines and the correction of the anterior crossbite in this compensatory treatment where the sagittal relationship was maintained but the maxillary incisors where protruded (1.NA = 33°, 1-NA = 8 mm), Table 1.

The correction of pseudo-Class III malocclusion with the use of a 2 × 4 appliance has been successful in nearly 100% of the cases without requiring a second phase for positive* overjet *to be obtained after treatment. This success is due to the advancement of point A, which remains stable over the long term [6], but it is important to remember that these patients maintain their facial character and tendency of growth, resembling to nontreated ones [4]. In our case the patient had cephalometric values of a skeletal Class I relationship (ANB = 1°) which was not reflected on her face; she maintained a Class III profile.

Correctly diagnosing a pseudo-Class III malocclusion makes a difference in the orthodontic treatment plan. The association of maxillary expansion and a 2 × 4 appliance can be successful during the correction of anterior crossbites.

## Figures and Tables

**Table 1:** Pre- and posttreatment measurements.

Measurements	Pretreatment	Posttreatment
SNA angle (°)	78	81
SNB angle (°)	76	79
ANB angle (°)	2	2
1-NA (mm)	1	8
1-NA (°)	18	33
1-NB (mm)	6	6
1-NB (°)	35	30
IMPA (°)	99	94
1-APo (mm)	5	4
Interincisal angle (°)	127	116
GoGn-SN (°)	40	35
*Y*-axis (°)	58	58
FMA (°)	29	28
Facial angle (°)	87	91
Convexity angle (°)	2	2
Upper lip-E line (mm)	0	−1
Lower lip-E line (mm)	3	4
